# ASO Author Reflections: Navigating the Nuances of Lymphedema Prevention with Immediate Lymphatic Reconstruction

**DOI:** 10.1245/s10434-025-17344-3

**Published:** 2025-04-20

**Authors:** Abbas M. Hassan, Carla S. Fisher, Aladdin H. Hassanein

**Affiliations:** Division of Plastic Surgery and Division of Breast Surgery, Department of Surgery, Indiana University School of Medicine, Indianapolis, IN USA

## Past

Breast cancer-related lymphedema (BCRL) following axillary lymph node dissection (ALND) remains a significant problem impacting quality of life.^1^ Historically, management focused on treatment after onset, but immediate lymphatic reconstruction (ILR), performed concurrently with ALND via lymphovenous anastomosis, emerged as a prophylactic strategy. Early ILR evidence was encouraging but was limited by small studies, heterogenous methodologies, short follow-up, and inconsistent BCRL definitions, leaving long-term efficacy unclear.^2^^,^^3^

## Present

Our study contributes a large series of 172 patients undergoing ILR, finding a cumulative BCRL incidence of 7.0% over a mean follow-up of approximately 23 months.^4^ This rate is substantially lower than historical ALND-only estimates (16.5–23.6%),^1^ supporting ILR’s preventative potential. Our analysis also highlighted a significant disparity: Black/African American patients experienced a disproportionately higher BCRL risk despite ILR.

## Future

To establish ILR’s definitive efficacy, future efforts must focus on several areas. Developing and consistently applying standardized BCRL diagnostic criteria—incorporating objective measures (tape, bioimpedance, perometry, lymphoscintigraphy) with subjective assessments and patient-reported outcomes—is crucial. Clarifying the clinical significance of abnormal lymphoscintigraphy findings in asymptomatic patients also warrants attention.^5^ Longer follow-up periods (>3 years) are necessary as lymphedema can manifest late. Variations in surgical techniques, such as using loupe magnification versus microsurgery or the necessity of vein grafting, require systematic evaluation, with insights expected from ongoing prospective studies.^3^ Finally, the alarming racial disparity observed requires investigation into underlying biological factors and potential healthcare inequities to develop targeted interventions for high-risk populations. Addressing these multifaceted challenges will clarify ILR’s precise role and help optimize BCRL prevention strategies.
